# The roles of prostaglandin F_2_ in regulating the expression of matrix metalloproteinase-12 via an insulin growth factor-2-dependent mechanism in sheared chondrocytes

**DOI:** 10.1038/s41392-018-0029-2

**Published:** 2018-11-23

**Authors:** Pei-Pei Guan, Wei-Yan Ding, Pu Wang

**Affiliations:** College of Life and Health Sciences, Northeastern University, Shenyang, 110819 P. R. China

## Abstract

Osteoarthritis (OA) was recently identified as being regulated by the induction of cyclooxygenase-2 (COX-2) in response to high fluid shear stress. Although the metabolic products of COX-2, including prostaglandin (PG)E_2_, 15-deoxy-Δ^12,14^-PGJ_2_ (15d-PGJ_2_), and PGF_2α_, have been reported to be effective in regulating the occurrence and development of OA by activating matrix metalloproteinases (MMPs), the roles of PGF_2α_ in OA are largely overlooked. Thus, we showed that high fluid shear stress induced the mRNA expression of MMP-12 via cyclic (c)AMP- and PGF_2α_-dependent signaling pathways. Specifically, we found that high fluid shear stress (20 dyn/cm^2^) significantly increased the expression of MMP-12 at 6 h ( > fivefold), which then slightly decreased until 48 h ( > threefold). In addition, shear stress enhanced the rapid synthesis of PGE_2_ and PGF_2α_, which generated synergistic effects on the expression of MMP-12 via EP2/EP3-, PGF2α receptor (FPR)-, cAMP- and insulin growth factor-2 (IGF-2)-dependent phosphatidylinositide 3-kinase (PI3-K)/protein kinase B (AKT), c-Jun N-terminal kinase (JNK)/c-Jun, and nuclear factor kappa-light-chain-enhancer of activated B cells (NF-κB)-activating pathways. Prolonged shear stress induced the synthesis of 15d-PGJ_2_, which is responsible for suppressing the high levels of MMP-12 at 48 h. These in vitro observations were further validated by in vivo experiments to evaluate the mechanisms of MMP-12 upregulation during the onset of OA by high fluid shear stress. By delineating this signaling pathway, our data provide a targeted therapeutic basis for combating OA.

## Osteoarthritis: Shear stress triggers cartilage-destroying molecular pathway

Shear stress induces cartilage cells to produce hormone-like molecules that activate the expression of an enzyme implicated in the development of osteoarthritis, a degenerative joint disease. Pu Wang and colleagues from Northeastern University in Shenyang, China, exposed human cartilage cells to high fluid shear stress for up to 2 days. This frictional strain rapidly stimulated the production of a proinflammatory enzyme, COX-2, which in turn promoted the synthesis of two hormone-like substances, called prostaglandins. These prostaglandins, PGE_2_ and PGF_2α_, then induced expression of an osteoarthritis-associated enzyme called MMP-12 that destroys the supporting structure that surrounds cartilage cells. The researchers, working both in human cells and in mouse models, further delineated several intermediate signaling molecules in the pathway linking shear stress with MMP-12 activation, thereby revealing several new potential drug targets for combating osteoarthritis in patients.

## Introduction

Recent studies have changed the traditional concept of osteoarthritis (OA) from a prototypical noninflammatory arthropathy to an inflammatory component.^1^ Many investigations have demonstrated that OA is associated with proinflammatory factors, including biomechanical stress, matrix-degrading enzymes, nitric oxide, and cytokine reactive oxygen species (ROS).^1^ Among these factors, fluid shear stress induced by mechanical loading has been identified to be critical for causing the pathogenesis of OA by precipitating irreversible cartilage erosion.^2^ A series of our investigations sustain the fact that high fluid shear stress induced the activity of cyclooxygenase-2 (COX-2), which is involved in the occurrence and development of OA.^3–6^ By inducing the expression of prostaglandins (PGs), matrix metalloproteinases (MMPs), and proinflammatory cytokines, high fluid shear stress has been reported to be critical for mediating the exacerbation of OA by COX-2.^7,8^ The antagonistic effects of 15-deoxy-Δ^12,14^-PGJ_2_ (15d-PGJ_2_) and PGE_2_ in regulating the temporal synthesis of MMP-9 were reported in detail in our previous studies.^8^ In addition, PGE_2_ and 15d-PGJ_2_ have been reported to have synergistic effects in stimulating the expression of MMP-1 in sheared chondrocytes.^9^ Apart from PGE_2_ and 15d-PGJ_2_, proinflammatory cytokines, such as interleukin-1β (IL-1β) and fibroblast growth factor-2 (FGF-2), have been shown to be responsible for regulating the enzymatic activity of MMP-9 and MMP-1 in shear-stimulated human chondrocytes.^8,9^ Although the detailed mechanisms are not thoroughly characterized, it has been indicated that the progressive erosion of cartilage involves the actions of COX-2 and its metabolic products (i.e., PGs), as well as secreted cytokines, such as IL-1β and tumor necrosis factor-α (TNF-α), thus leading to the induction of the expression of matrix-degrading enzymes, such as MMPs.^10^

Although PGE_2_ and 15d-PGJ_2_ are reported to be critical for causing OA by modulating the expression of MMP-1 and MMP-9,^8,9^ the roles of PGF_2α_ in the pathogenesis of OA via regulating MMP activities have been highly overlooked. Prior studies have demonstrated that PGF_2α_ was mainly derived from COX-1.^11,12^ In addition, COX-2 induced by laminar shear stress is responsible for the formation of PGF_2α_ in the human umbilical cord endothelial cells.^13^ The initial roles of PGF_2α_ in inflammation were identified in vitro and in vivo by the administration of nonsteroidal antiinflammatory drugs (NSAIDs), such as ibuprofen.^12^ In agreement with this observation, larger quantities of 15-keto-dihydro-PGF_2α_, a stable metabolite of PGF_2α_ that reflects in vivo PGF_2α_ biosynthesis, have been identified in acute and chronic inflammation situations.^14^ Furthermore, elevated biosynthesis of PGF_2α_ has been reported in patients suffering from rheumatoid arthritis, psoriatic arthritis, reactive arthritis, and osteoarthritis.^15^ Moreover, PGF_2α_ receptors (FPRs) have been indicated to mediate the effects of PGF_2α_ on the pathogenesis of inflammation in lipopolysaccharide (LPS)-induced tachycardia^16^ and pulmonary fibrosis.^17^

The emerging roles of PGF_2α_ in acute and chronic inflammation suggest that it may regulate the occurrence and development of OA by increasing the activity of MMPs. For example, PGF_2α_ can induce the enzymatic activity of MMP-2 in human ciliary muscle cells.^18^ In addition, the acute roles of PGF_2α_ in the secretion of MMP-2 were further confirmed in human ciliary muscle cells.^19^ Although there is no other evidence showing a regulatory relationship between PGF_2α_ and MMPs, several MMPs, such as MMP-1, −2, −3, −9, −12, and −13, have been reported to be upregulated in the subchondral bone of OA rats.^7^ Although the activation of MMPs is believed to impair the cartilage by degrading the extracellular matrix (ECM), their substrates for degradation are thoroughly different. For example, both MMP-3 and MMP-13 have the ability to degrade type II collagen, which potentially contributes to OA.^20^ Moreover, aggrecan, another important collagenase, is also the substrate of MMP-3. In addition to these collagenases, MMP-2 and MMP-9 are produced by chondrocytes as proteinases, which may have roles in the further degradation of various matrix components.^21^ MMP-12 has numerous substrates, such as elastin.^22^ MMP-12 expression was markedly increased with the age and body mass index of OA patients.^23^ MMP-12 expression was also upregulated in the Zn^2+^-ZIP8-MTF1-regulated pathogenesis of OA.^24^ However, the regulating mechanisms of MMP-12 upregulation are highly overlooked during the development and progression of OA.

Due to the important roles of MMP-12 in the occurrence and progression of OA, we sought to elucidate the mechanisms of COX-2 in upregulating the expression of MMP-12 via its metabolic products, including PGE_2_ and PGF_2α_, in shear-activated human chondrocytes. Specifically, we demonstrate that high fluid shear stress markedly induces MMP-12 expression. In addition, we show shear-induced endogenous PGE_2_ and PGF_2α_ have synergistic effects in upregulating the synthesis of MMP-12 in human chondrocytes. Furthermore, we demonstrate the key roles of cyclic (c)AMP and IGF-2, which are downstream of PGE_2_ and PGF_2α_ in the regulation of MMP-12 via the phosphatidylinositide 3-kinase (PI3-K)/protein kinase B (AKT), c-Jun N-terminal kinase (JNK)/c-Jun and nuclear factor kappa-light-chain-enhancer of the activated B cells (NF-κB) pathways.

## Materials and methods

### Reagents

NS398, PGE_2_, PGF_2α_, forskolin, LY294002, SP600125, and GW9662 were purchased from Sigma-Aldrich (St. Louis, MO, USA). AH6809, Sulprostone, 6-amino-4-(4-phenoxyphenylethylamino) quinazoline (QNZ), and 15-deoxy-Δ^12,14^-PGJ_2_ (15d-PGJ_2_) were purchased from Enzo Life Sciences (Shanghai, China). Human recombinant IGF-2 was purchased from R&D systems (Shanghai, China). Antibodies specific for p-p65 (Ser 536), p65, Akt, p-Akt (Ser 473), c-Jun, p-c-Jun (Ser 63), and β-actin were purchased from Cell Signaling Technology (Danvers, MA, USA). PGF_2α_ receptor, mPGES-1, PGFS, scramble siRNAs, and antibodies specific for MMP-12 and MMP-11 were purchased from Santa Cruz Biotechnology, Inc. (Santa Cruz, CA, USA). The PGE_2_ and cAMP enzyme-linked immunosorbant assay kits were obtained from Cayman Chemical Company (Ann Arbor, MI, USA), and the 15d-PGJ_2_ EIA kit was purchased from Assay Designs, Inc. (Ann Arbor, MI, USA). The PGF_2α_ and IGF-2 enzyme immunoassay kits were obtained from Oxford Biomedical Research (Beijing, China) and R&D systems (Shanghai, China), respectively. The chromatin immunoprecipitation (ChIP) EZ-ChIP kit was obtained from Upstate Biotechnology. The dual-luciferase reporter assay kit was obtained from Promega (Madison, WI, USA). All reagents for qRT-PCR and SDS-PAGE experiments were obtained from Bio-Rad Laboratories, Inc. (Shanghai, China). All other reagents were obtained from Invitrogen (Carlsbad, CA, USA), unless otherwise specified.

### Shear stress exposure and cell culture

Human T/C-28a2 chondrocytes (Gifted by Dr. Goldring) were seeded and cultured on glass slides in DMEM/F12 medium supplemented with 1 × 10^−1^ FBS under the condition of 37 °C in 5% CO_2_ as previously described.^5,6,8,25^ Prior to exposure in high fluid shear stress, cells were cultured in serum-free medium supplemented with 1 × 10^−2^ (ml:ml) Nutridoma-SP (Roche Applied Science; Indianapolis, IN, USA) for 18 h, an alternative replacement of serum, which established quiescence in the monolayer and maintained the chondrocytic phenotype.^5,6,8^ Cells were then exposed to a shear stress with the strength of 20 dyn/cm^2^ for up to 48 h in medium that contained 1% Nutridoma-SP, using a streamer gold flow device (Flexcell International, Hillsborough, NC). In the treatment of pharmacological agents, the indicated concentrations of the chemicals were added to the medium immediately prior to the onset of shear exposure. In the static experiments, T/C-28a2 chondrocytes were seeded on 6 cm tissue culture dishes (10^6^ cells per dish) in DMEM/F12 medium supplemented with 10% FBS.^3,4^ After 24 h, T/C-28a2 cells were grown in serum-free medium for an additional 24 h prior to being incubated with specific pharmacological inhibitors in the presence or absence of exogenously added IGF-2.^3,4^

### Transient transfection

Human T/C-28a2 chondrocytes were transfected with 100 nM of a siRNA oligonucleotide sequence that targeted mPGES-1 or PGFS. In the control experiments, 100 nM of scramble siRNA were transfected to human T/C-28a2 chondrocytes. In the promoter assays, 1.6 μg/slide of the MMP-12 promoter reporter construct along with the pRL-SV40 vector were transfected to human T/C-28a2 cells. Transfected cells were allowed to recover in growth medium for at least 12 h and were then incubated overnight in medium that contained 1% Nutridoma-SP prior to exposure to high fluid shear stress.

### Quantitative real-time PCR (qRT-PCR)

qRT-PCR assays were performed on the iCycler iQ detection system (Bio-Rad) with total RNA, using the iScript one-step RT-PCR kit with SYBR green (Bio-Rad) and primers. The GenBank accession numbers and forward (F-) and reverse (R-) primers are as follows:

PGFS (NM_003739.5), F- TCTCTGTACCACCTGGGAGG,

R- GGTCCACCCATCGTTTGTCT;

IGF-2 (NM_000612), F- ACAGCTGACCTCATTTCCCG,

R- TCCCATTGGTGTCTGGAAGC.

The GenBank accession numbers and forward (F-) and reverse (R-) primers for mPGES-1, MMP-12 and GAPDH are provided in our previous publications.^6,26^ GAPDH was used as an internal control. Reaction mixtures were incubated at 50 °C for 15 min followed by 95 °C for 5 min, and 35 PCR cycles were subsequently performed with the following temperature profile: 95 °C 15 s, 58 °C 30 s, 68 °C 1 min, and 77 °C 20 s. Data were collected at the (77 °C 20 s) step to remove the potential fluorescent contribution from dimer-primers.^27,28^ The gene expression values were normalized to GAPDH. The ratio of MMP-12 or other gene mRNA expression was calculated via the following equation:

$${\mathrm{Ratio}} = \frac{{2^{\Delta Ct\,(MMP {\hbox{-}} 12_{Control} - MMP {\hbox{-}} 12_{Treatment})}}}{{2^{\Delta Ct\,(GAPDH_{Control} - GAPDH_{Treatment})}}}$$

### Western blot analysis

Human T/C-28a2 chondrocytes, from static and sheared cells, were lysed in radioimmune precipitation assay buffer [25 mM Tris•HCl pH 7.6, 150 mM NaCl, 1% NP-40 (g: ml), 1% sodium deoxycholate (g: ml), 0.1% SDS (g: ml)] supplemented with a proteinase inhibitor cocktail (Pierce Chemical Company). The protein concentration of the lysed cells was determined using bicinchoninic acid (BCA) protein assay reagent (Pierce Chemical Company). Four micrograms of total cell lysates was subjected to SDS-PAGE, transferred to a PVDF membrane and immunostained with a panel of specific antibodies. Each membrane was only probed using one antibody. β-actin was used as the loading control. All Western hybridizations were performed at least in triplicate using a different cell preparation each time.

### Measurement of IGF-2, PGE_2_, PGF_2α_, cAMP, and 15d-PGJ_2_ concentrations

The levels of IGF-2, PGE_2_, PGF_2α_, cAMP, and 15d-PGJ_2_ in the medium of the static and sheared cells or tissue extractions were determined using an ELISA kit following the manufacturer’s instructions. Briefly, 96-well plates were precoated with an IgG antibody. The wells were incubated with standards or test samples, following which an alkaline phosphatase (AP) conjugated-IGF-2, PGE_2_, PGF_2α_, cAMP, and 15d-PGJ_2_ antibody was added. AP subsequently catalyzed the pNpp substrate to produce a yellow color after the incubation, and the excess reagents are washed away. The optical intensity of the yellow coloration is reversely proportional to the amount of IGF-2, PGE_2_, PGF_2α_, cAMP, and 15d-PGJ_2_ captured in the plate. To further normalize the concentration of IGF-2, PGE_2_, PGF_2α_, cAMP, and 15d-PGJ_2_, the total protein in the medium or tissue extraction was measured by BCA kits. The concentration of total protein in the medium or tissue extraction was used as the loading control, and the results were expressed as IGF-2, PGE_2_, PGF_2α_, cAMP, or 15d-PGJ_2_ /total protein.

The methods for Luciferase promoter constructs, Promoter activity assays, EMSA, ChIP assay, Transgenic mice, Immunohistochemistry (IHC), and Animal committee were delineated and provided in the Supplementary Information.

### Statistics

Data represent the mean ± SE of at least three independent experiments. The statistical significance of differences between means was determined by Student’s *t*-test or one-way ANOVA, when appropriate. If means were shown to be significantly different, then multiple comparisons by pairs were performed using the Tukey test.^5,6,8^

## Results

### Shear-induced COX-2 is responsible for the synthesis of PGE_2_ and PGF_2α_, as well as the expression of IGF-2 and MMP-12 in human chondrocytes

**Fig. 1:**
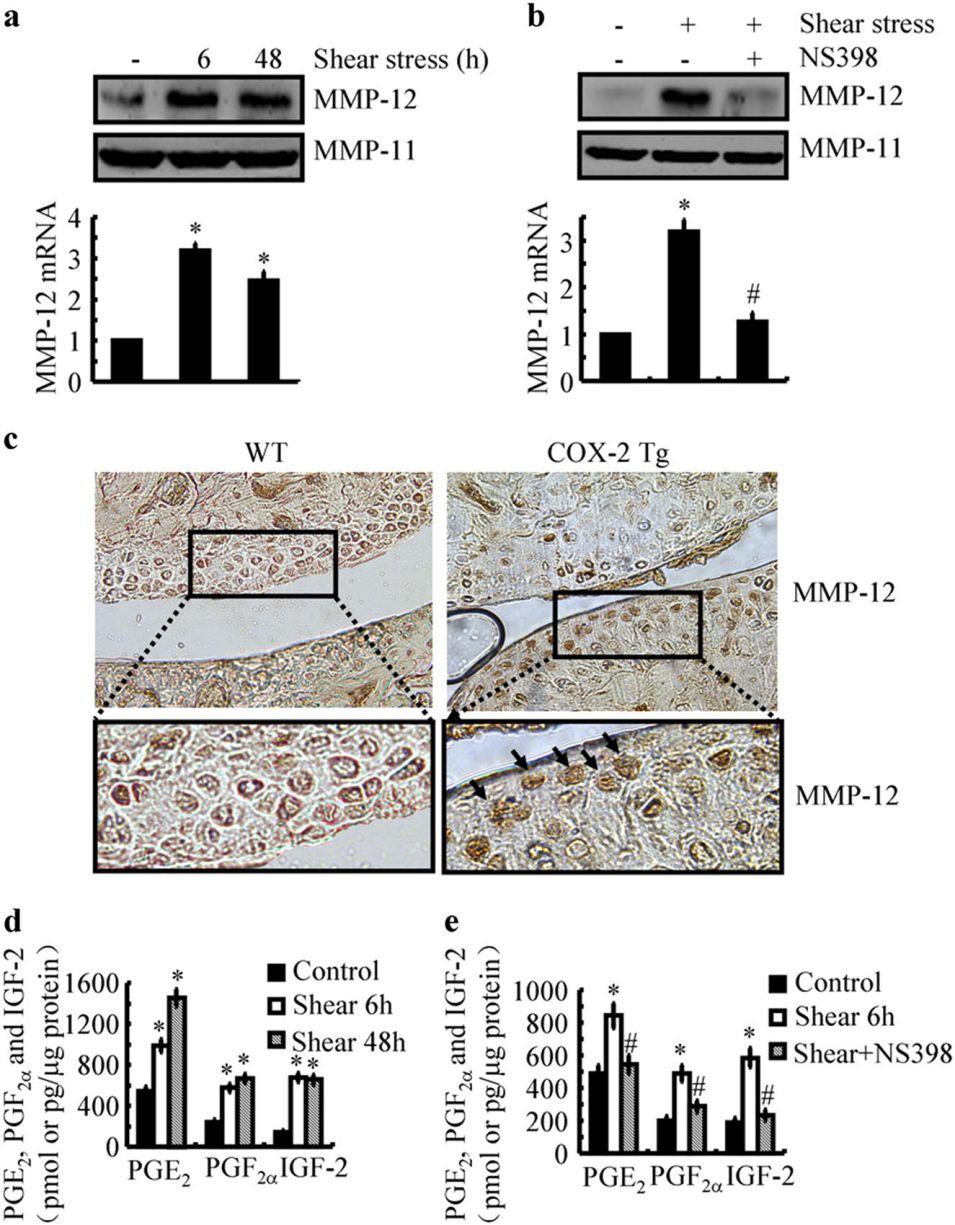
High fluid shear stress induces the progressive upregulation of MMP-12 and synthesis of PGE_2_, PGF_2α_, and IGF-2 in human chondrocytes. **a** and **d** T/C-28a2 chondrocytes were subjected to fluid shear stress (20 dyn/cm^2^) or static conditions (0 dyn/cm^2^) for the indicated time intervals. **b** and **e** In select experiments, T/C-28a2 chondrocytes were subjected to fluid shear stress (20 dyn/cm^2^) or static conditions (0 dyn/cm^2^) in the absence or presence of NS398 (10 μM) for 6 h. **a** and **b** MMP-12 mRNA and protein levels were determined by qRT-PCR and western blots, respectively. GAPDH and MMP-11 served as internal controls in qRT-PCR and western blots, respectively. (**c**) The slices of articular cartilage were prepared and immunostained with MMP-12 antibody. **d** and **e** The secretions of IGF-2, PGE_2_, and PGF_2α_ were determined by enzyme immunoassay kits. Total protein in the medium served as an internal control in enzyme immunoassay. Data represent the mean ± SE of at least three independent experiments. *p *<* *0.05* with respect to static control. ^#^ *p* < 0.05 with respect to shear stress treatment

Our previous studies have indicated that continuous exposure of human T/C-28a2 chondrocytes to high fluid shear stress (20 dyn/cm^2^) induced the fast expression of COX-2 at 2 h, which maintained high levels of COX-2 until 48 h.^9^ Interestingly, the protein synthesis of MMP-12 was markedly upregulated at 6 h following shear stress exposure, which decreased slightly until 48 h in T/C-28a2 cells (Fig. 1a). To further confirm the key roles of COX-2 in MMP-12 regulation, human T/C-28a2 cells were exposed to fluid shear stress (20 dyn/cm^2^) for 48 h in the absence or presence of a COX-2 inhibitor, NS398 (10 μM). The results showed that NS398 (10 μM) treatment blocked the effects of fluid shear stress on inducing MMP-12 expression by suppressing the activity but not the synthesis of COX-2 (Fig. 1b).^9^ In agreement with these in vitro observations, the in vivo results demonstrated that MMP-12 expression was upregulated in COX-2 Tg mice (Fig. 1c). We, therefore, conclude that COX-2 played key roles in mediating the effects of shear stress on MMP-12 expression.

In view of the potential contributions of IGF-2 and PGs in regulating the activity of MMP-12,^26^ we first determined the production of PGE_2_/F_2α_ and the expression of IGF-2. As shown in Fig. 1d, 20 dyn/cm^2^ of shear stress induced the production of PGE_2_/F_2α_ and the expression of IGF-2 in human T/C-28a2 cells. In addition, inhibition of COX-2 activity significantly reversed both the production of PGE_2_/F_2α_ and the expression of IGF-2 in sheared-T/C-28a2 cells (Fig. 1e). The efficacy of NS398 in suppressing the production of PGs and the expression of IGF-2 demonstrated the pivotal roles of COX-2 in regulating the activity of MMP-12 in human chondrocytes.

### Both PGE_2_ and PGF_2α_ are responsible for upregulating the expression of MMP-12 in shear-stimulated chondrocytes

**Fig. 2:**
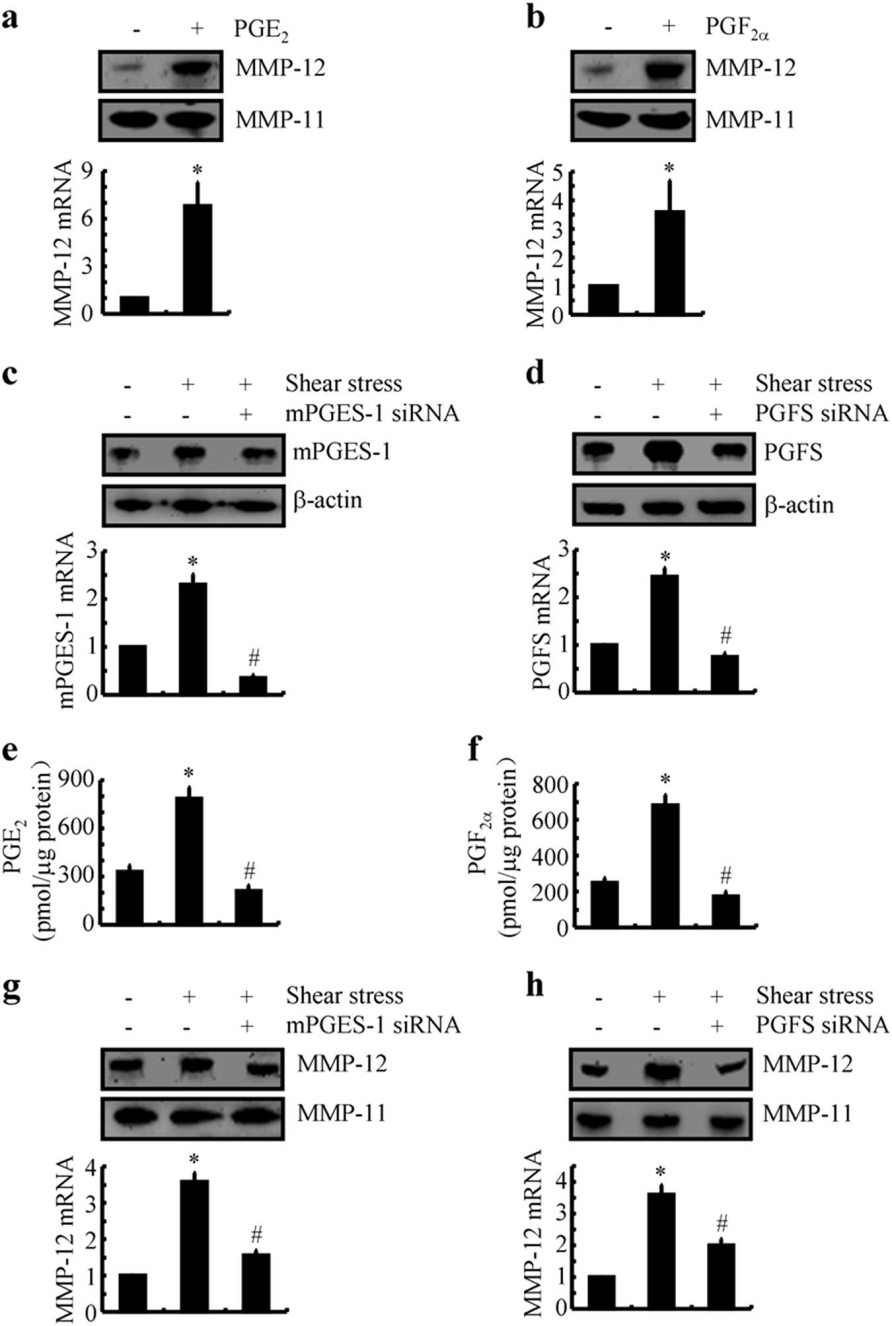
Involvement of PGE_2_ and PGF_2α_ in upregulating the expression of MMP-12 in shear-activated human chondrocytes. T/C-28a2 chondrocytes were treated with the indicated concentrations of exogenous PGE_2_ (10 μM) (**a**) or PGF_2α_ (10 μM) (**b**) for 48 h. In select experiments, T/C-28a2 chondrocytes were transfected with a siRNA specific target mPGES-1 (**c**, **e**, **g**) or PGFS (**d**, **f**, **h**) before subjecting to fluid shear stress (20 dyn/cm^2^) or static conditions (0 dyn/cm^2^). **a**, **b**, **g**, **h** MMP-12 mRNA and protein levels were determined by qRT-PCR and western blots, respectively. GAPDH and MMP-11 served as internal controls in qRT-PCR and western blots, respectively. **c** and **d** mPGES-1 or PGFS expression levels were determined by qRT-PCR and western blots, respectively. GAPDH and β-actin served as internal controls in qRT-PCR and western blots, respectively. **e** and **f** The secretions of PGE_2_ and PGF_2α_ were determined using enzyme immunoassay kits. Total protein in the medium served as an internal control in enzyme immunoassays. Data represent the mean ± S.E. of at least three independent experiments. *p *<* *0.05* with respect to static control. ^#^ *p* < 0.05 with respect to shear stress treatment

In light of the critical roles of COX-2 in regulating the expression of MMP-12, we subsequently determined the effects of PGE_2_ and PGF_2α_ on the expression of MMP-12 in sheared-activated T/C-28a2 cells. To verify this hypothesis, experiments were carried out to treat T/C-28a2 cells with PGE_2_ or PGF_2α_. The results demonstrated that PGE_2_ (10 μM) and PGF_2α_ (10 μM) treatment increased the expression of MMP-12 in human T/C-28a2 cells (Fig. 2a, b). To further validate these observations, siRNAs specific for mPGES-1 or PGFS were used to knockdown the corresponding gene expression. The effectiveness of this genetic intervention was confirmed at the mRNA and protein levels, which, in turn, inhibited the expression of shear-induced mPGES-1 and PGFS (Fig. 2c, d). Because mPGES-1 and PGFS are the enzymes for the biosynthesis of PGE_2_ and PGF_2α_, we continued to evaluate the effects of siRNAs on the production of PGE_2_ and PGF_2α_. As expected, the inductions of PGE_2_ and PGF_2α_ were also attenuated by the transfection of T/C-28a2 cells with mPGES-1 or PGFS siRNA (Fig. 2e, f). Both mPGES-1 and PGFS knockdown inhibited the shear-induced MMP-12 upregulation at 2 h without affecting the MMP-11 basal expression level (Fig. 2g, h). Therefore, PGE_2_ and PGF_2_ exhibited synergistic effects on shear-stimulated MMP-12 expression in human chondrocytes.

### PGE_2_ and PGF_2α_ stimulate the expression of MMP-12 via cAMP- and IGF-2-dependent mechanisms in shear-activated human chondrocytes

**Fig. 3:**
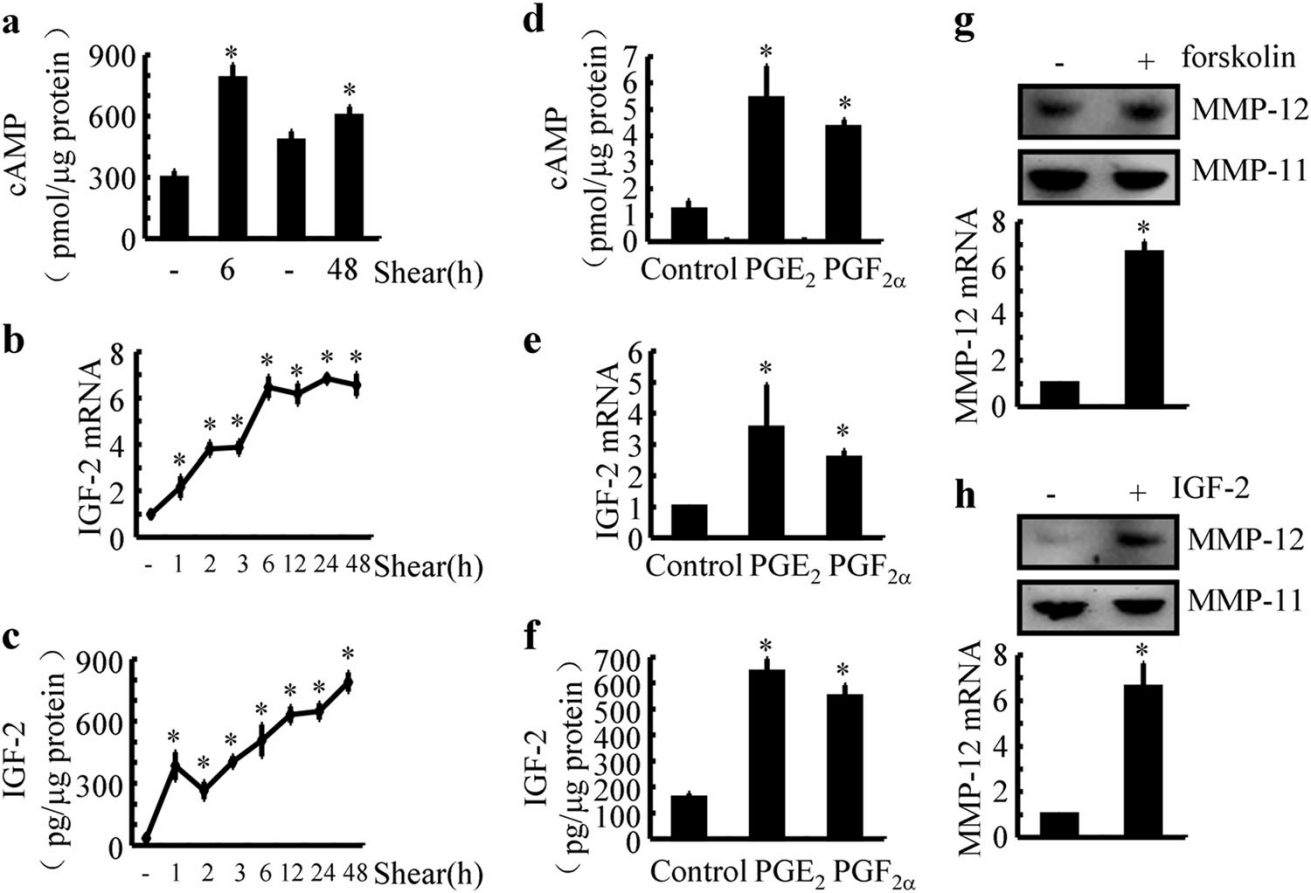
Shear-induced cAMP and IGF-2 are responsible for the synthesis of MMP-12 in human chondrocytes. **a**–**c** T/C-28a2 chondrocytes were subjected to fluid shear stress (20 dyn/cm^2^) or static conditions (0 dyn/cm^2^) for the indicated time intervals. **d**–**f** In select experiments, T/C-28a2 chondrocytes were treated with the indicated concentrations of exogenous PGE_2_ (10 μM) or PGF_2α_ (10 μM). **g** and **h** In separate experiments, cells were incubated with forskolin (10 μM) or IGF-2 (10 ng/ml) for 48 h. **a**, **c**, **d**, **f** The synthesis of cAMP and IGF-2 was determined by enzyme immunoassay kits. Total protein served as the internal control. **b** and **e** mRNA expression of IGF-2 was determined by qRT-PCR, and GAPDH served as the internal control. **g** and **h** MMP-12 mRNA and protein levels were determined by qRT-PCR and western blots, respectively. GAPDH and MMP-11 served as internal controls in qRT-PCR and western blots, respectively. Data represent the mean ± SE of at least three independent experiments. *p *<* *0.05* with respect to static control

We subsequently aimed to elucidate the mechanisms of MMP-12 synthesis in sheared chondrocytes. In light of the potential roles of cAMP and IGF-2 in shear-induced MMP-12 expression,^26^ we first determined the production of cAMP and IGF-2 in shear-stimulated human chondrocytes. Our analysis indicates that high fluid shear stress rapidly stimulated the production of cAMP, which was sustained above basal level until 48 h of shear stress exposure (Fig. 3a). In addition, the mRNA and protein expression of IGF-2 remained at high levels after stimulation by high fluid shear stress at 2 h (Fig. 3b, c). In view of these observations, it is reasonable to speculate whether PGE_2_ and PGF_2α_ have the ability to mediate the effects of high fluid shear stress on stimulating the production of cAMP and the expression of IGF-2 in T/C-28a2 cells. As expected, PGE_2_ (10 μM) and PGF_2α_ (10 μM) treatment clearly induced the production of cAMP and the expression of IGF-2 in human T/C-28a2 chondrocytes (Fig. 3d–f). To further elucidate the effects of cAMP and IGF-2 on the expression of MMP-12, we further treated T/C-28a2 cells with forskolin (10 μM), an activator of cAMP, or IGF-2 (10 ng/ml) for 48 h. The results demonstrated that forskolin and IGF-2 treatment significantly increased the expression of MMP-12 in human chondrocytic cells (Fig. 3g, h). These results demonstrated that cAMP and IGF-2 mediated the effects of PGE_2_ and PGF_2α_ on stimulating MMP-12 expression in shear-activated human chondrocytes.

### Shear-stimulated the expression of MMP-12 via a PGE_2_-/EPs- and PGF_2α_-/FPR-dependent cAMP and IGF-2 induction mechanism

**Fig. 4:**
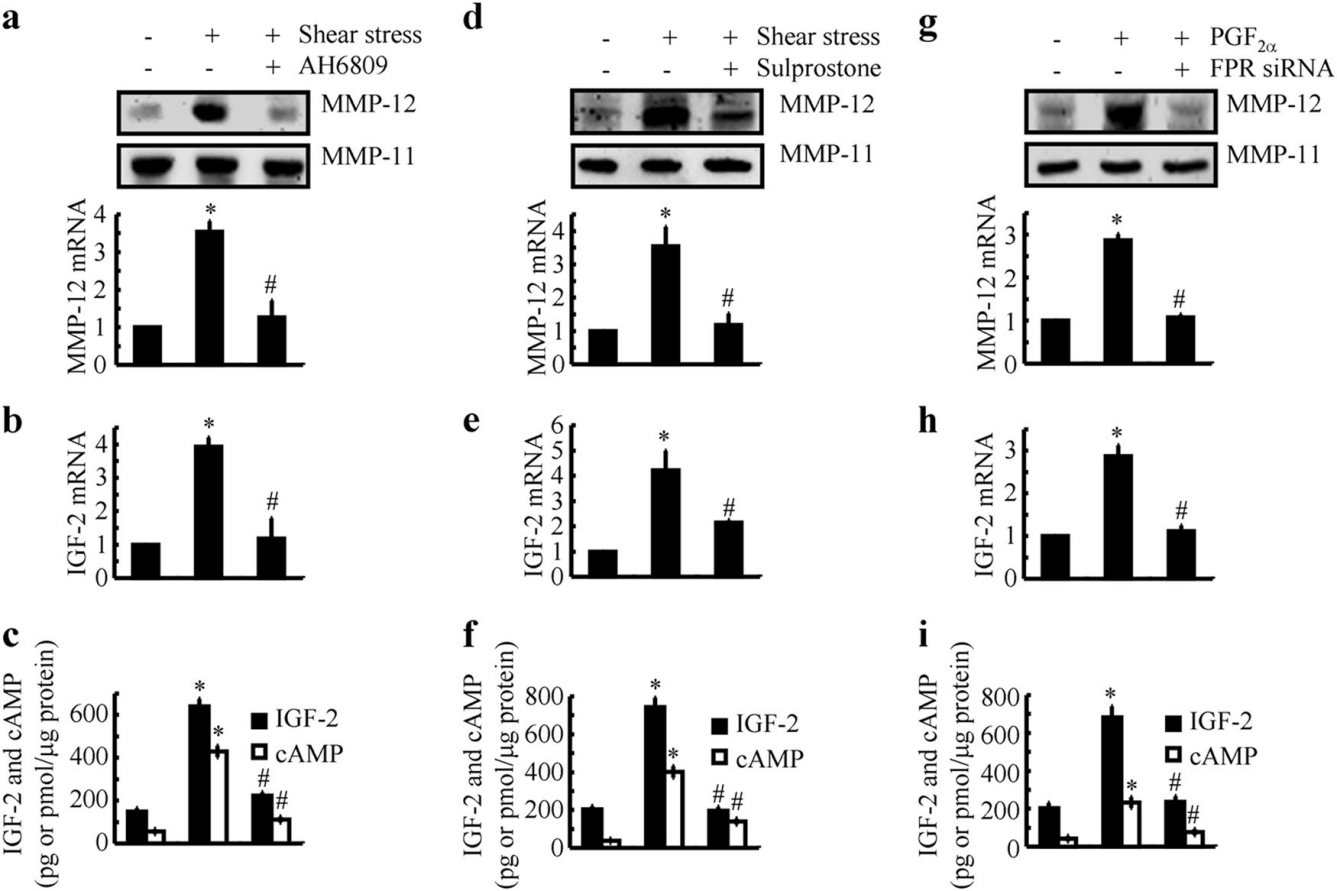
EP2/EP3 and FPR mediated the effects of PGE_2_ and PGF_2α_ on stimulating the expression of MMP-12 in shear-activated human T/C-28a2 chondrocytes. **a**–**f** T/C-28a2 chondrocytes were subjected to fluid shear stress (20 dyn/cm^2^) or static conditions (0 dyn/cm^2^) in the absence or presence of AH6809 (3 μM) or sulprostone (1 μM) for 6 h. **g**–**i** In select experiments, T/C-28a2 chondrocytes were treated with the indicated concentrations of exogenous PGF_2α_ (10 μM) in the absence or presence of FPR siRNA for 48 h. **a**, **d**, **g** MMP-12 mRNA and protein levels were determined by qRT-PCR and western blots, respectively. GAPDH and MMP-11 served as internal controls in qRT-PCR and western blots, respectively. **b**, **c**, **e**, **f**, **h**, **i** The mRNA and protein expression of IGF-2 were determined using qRT-PCR and an enzyme immunoassay kit, respectively. GAPDH and total protein in the medium served as internal controls in qRT-PCR and enzyme immunoassay, respectively. Data represent the mean ± SE of at least three independent experiments. *p *<* *0.05* with respect to static or vehicle-treated controls. ^#^ *p* < 0.05 with respect to shear stress or PGF_2α_ treatment

Our previous studies indicated that T/C-28a2 chondrocytes are enriched in EP2, EP3, and very low levels of EP4; however, they lack EP1 receptors. Moreover, the application of high fluid shears simultaneously upregulates EP2 and downregulates EP3 expression in human T/C-28a2 cells.^3,5^ In view of these findings, we investigated the effects of an EP2 receptor antagonist, AH6809, and an EP3 receptor agonist, sulprostone, on shear-induced MMP-12 expression. For their ability to suppress the synthesis of cAMP,^3,5^ AH6809 (3 μM), or sulprostone (1 μM) treatment suppressed the shear-stimulated MMP-12 mRNA and protein expression in human T/C-28a2 cells (Fig. 4a, d). Furthermore, AH6809 (3 μM) or sulprostone (1 μM) treatment abrogated the shear-induced cAMP and IGF-2 secretion in T/C-28a2 chondrocytes (Fig. 4b, c, e, f). On the basis of these observations, it is clear that shear-induced the expression of MMP-12 via the mechanisms of the PGE_2_-/EP2-/EP3-dependent cAMP and IGF-2 pathways.

Apart from PGE_2_ signals, PGF_2α_ also exerts its effects via its FPRs.^29^ To further identify the involvement of FPRs in regulating the expression of MMP-12, experiments were designed to treat human T/C-28a2 cells with PGF_2α_ (10 μM) in the absence or presence of AKR1C3 siRNA for 48 h. The results demonstrated that PGF_2α_ receptor (FPR) siRNA treatment clearly blocked the effects of PGF_2α_ on stimulating the mRNA and protein expression of IGF-2 in human T/C-28a2 cells (Fig. 4 h, i). As a consequence, the mRNA and protein expression of MMP-12 were also inhibited by FPR siRNA treatment in PGF_2_-stimulated T/C-18a2 cells (Fig. 4 g). Thus, it is clear that PGF_2α_ receptors are involved in the signals of PGF_2α_ to regulate MMP-12 expression by stimulating cAMP formation and IGF-2 expression in human chondrocytes.

### Involvement of the PI3-K/AKT-activated NF-κB and c-Jun signaling pathways in regulating MMP-12 expression by the stimulation of PGE_2_ and PGF_2α_

**Fig. 5:**
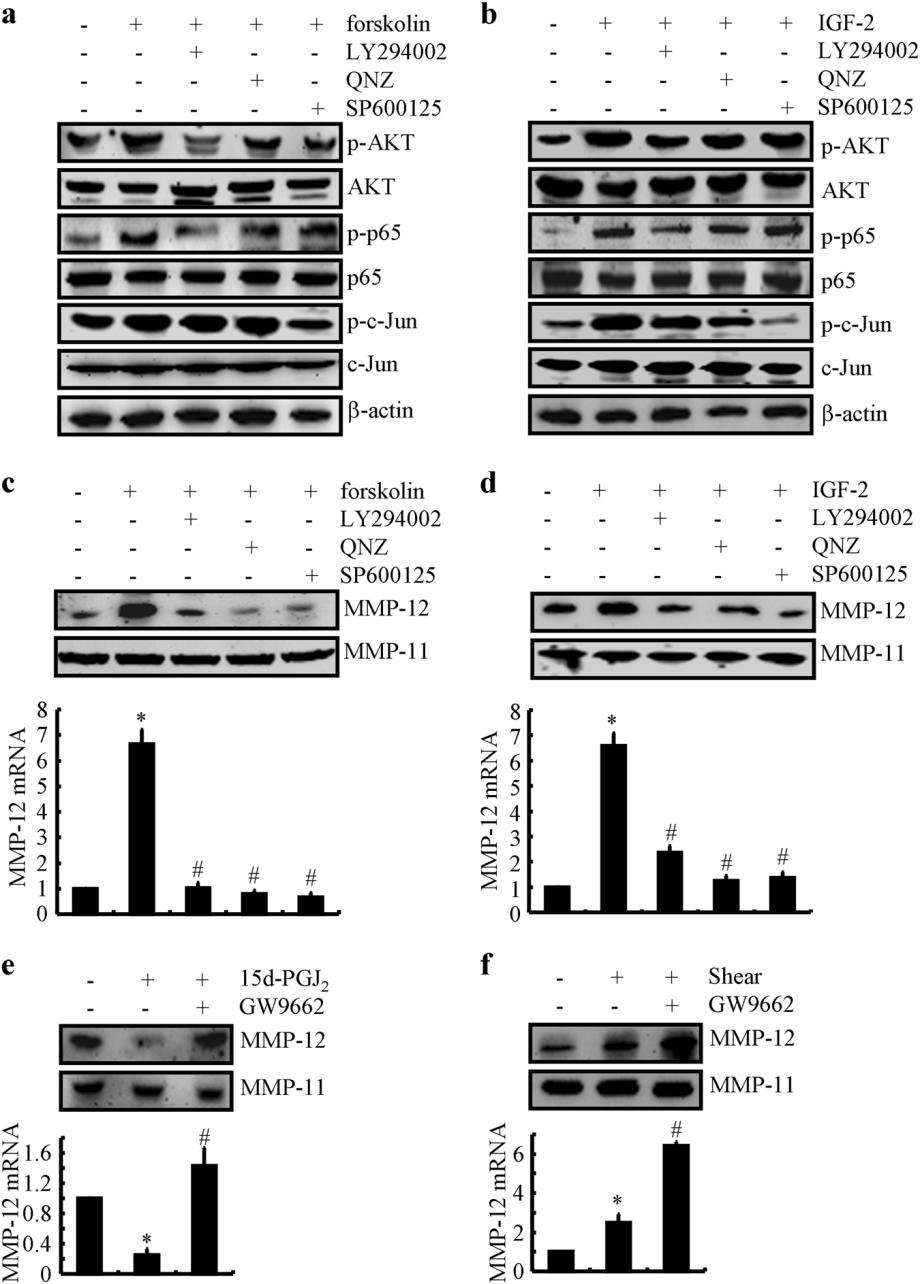
cAMP, IGF-2 and 15d-PGJ_2_ are critical for MMP-12 expression via PI3-K-dependent NF-κB-, c-Jun-, and PPARγ-activating mechanisms in human chondrocytes. T/C-28a2 chondrocytes were treated with (**a**, **c**) forskolin (10 μM) or (**b**, **d**) IGF-2 (10 ng/ml) in the absence or presence of the PI3-K inhibitor LY294002 (10 μM), NF-κB inhibitor QNZ (1 μM) or JNK inhibitor SP600125 (10 μM) for 48 h. In select experiments, (**e**) T/C-28a2 chondrocytes were treated with 15d-PGJ_2_ (500 nM) in the absence or presence of the PPARγ antagonist GW9662 (1 μM) for 48 h. **f** In separate experiments, T/C-28a2 chondrocytes were subjected to fluid shear stress (20 dyn/cm^2^) or static conditions (0 dyn/cm^2^) in the absence or presence of the PPARγ antagonist GW9662 (1 μM) for 48 h. **a** and **b** Phosphorylated Akt (Ser473), p65 (Ser 536), c-Jun (Ser 63), total Akt, p65, and c-Jun protein expressions are shown by immunoblotting using specific Abs. Equal loading in each lane is ensured by the similar intensities of β-actin. **c**–**f** MMP-12 mRNA and protein levels were determined by qRT-PCR and western blots, respectively. GAPDH and MMP-11 served as internal controls in qRT-PCR and western blots, respectively. Data represent the mean ± SE of at least three independent experiments. *p *<* *0.05* with respect to vehicle-treated control. ^#^ *p* < 0.05 with respect to forskolin, IGF-2 or 15d-PGJ_2_ treatment

In light of these observations, we continued to elucidate the signaling events of MMP-12 synthesis in shear-stimulated chondrocytes. Given the key roles of cAMP and IGF-2 in shear-activated MMP-12, we initially determined the mechanisms modulated by cAMP and IGF-2. As our previous studies showed that the PI3-K, NF-κB, and c-Jun pathways are stimulated by high fluid shear stress,^9^ we investigated their potential contribution to the expression of cAMP and IGF-2 in response to exogenously added forskolin and IGF-2. Forskolin (10 μM) and IGF-2 (10 ng/ml) rapidly stimulated the activities of AKT (Ser 473), NF-κB (Ser 536), and c-Jun (Ser 63) by phosphorylation without affecting the total AKT, NF-κB, and c-Jun levels, respectively, in T/C-28a2 cells (Fig. 5a, b), thereby suggesting the involvement of the PI3-K/AKT, NF-κB, and c-Jun pathways in modulating the MMP-12 expression in human chondrocytes. As expected, elevated levels of AKT, NF-κB, and c-Jun potentially contribute to the expression of MMP-12 in forskolin-activated and IGF-2-activated T/C-28a2 chondrocytes (Fig. 5c, d). To further validate these observations, we treated T/C-28a2 chondrocytes with forskolin and IGF-2 in the absence or presence of the specific PI3-K inhibitor LY294002 (10 μM), the NF-κB inhibitor QNZ (1 μM), or the JNK inhibitor SP600125 (10 μM). The results demonstrated that the treatment of T/C-28a2 chondrocytes with different inhibitors abolished forskolin-induced or IGF-2-induced AKT, NF-κB, and c-Jun phosphorylation and the MMP-12 mRNA and protein levels (Fig. 5c, d). Collectively, our data demonstrate that shear-stimulated MMP-12 expression is mediated via the synergistic actions of endogenously produced cAMP and IGF-2 and their downstream signals in PI3-K/AKT, NF-κB, and JNK-dependent manners.

### Activation of PPARγ is critical for suppressing the synthesis of MMP-12 in shear-stimulated human chondrocytes

In light of our results showing the induction of 15d-PGJ_2_ at 48 h of shear stress exposure,^6^ we continued to examine whether 15d-PGJ_2_ exerts its effects via its primary receptor PPARγ.^30^ As shown in Fig. 5e, exogenous 15d-PGJ_2_ slightly suppressed the expression of MMP-12 in human T/C-28a2 chondrocytes. To further identify the involvement of PPARγ in regulating the expression of MMP-12, experiments were carried out to treat human T/C-28a2 cells with 15d-PGJ_2_ in the absence or presence of the PPARγ-specific antagonist GW9662 (1 μM). The results demonstrated that GW9662 treatment clearly attenuated the effects of 15d-PGJ_2_ on suppressing the expression of MMP-12 in human chondrocytes (Fig. 5e). Interestingly, GW9662 (1 μM) addition stimulated the suppressive effects of 15d-PGJ_2_ on shear-stimulated MMP-12 expression in human chondrocytes (Fig. 5f). Taken together, our data suggest the key roles of PPARγ in downregulating the expression of MMP-12 in 15d-PGJ_2_-dependent shear-stimulated human T/C-28a2 chondrocytes.

### NF-κB p65 subunit and c-Jun are involved in shear-upregulated MMP-12 expression

**Fig. 6:**
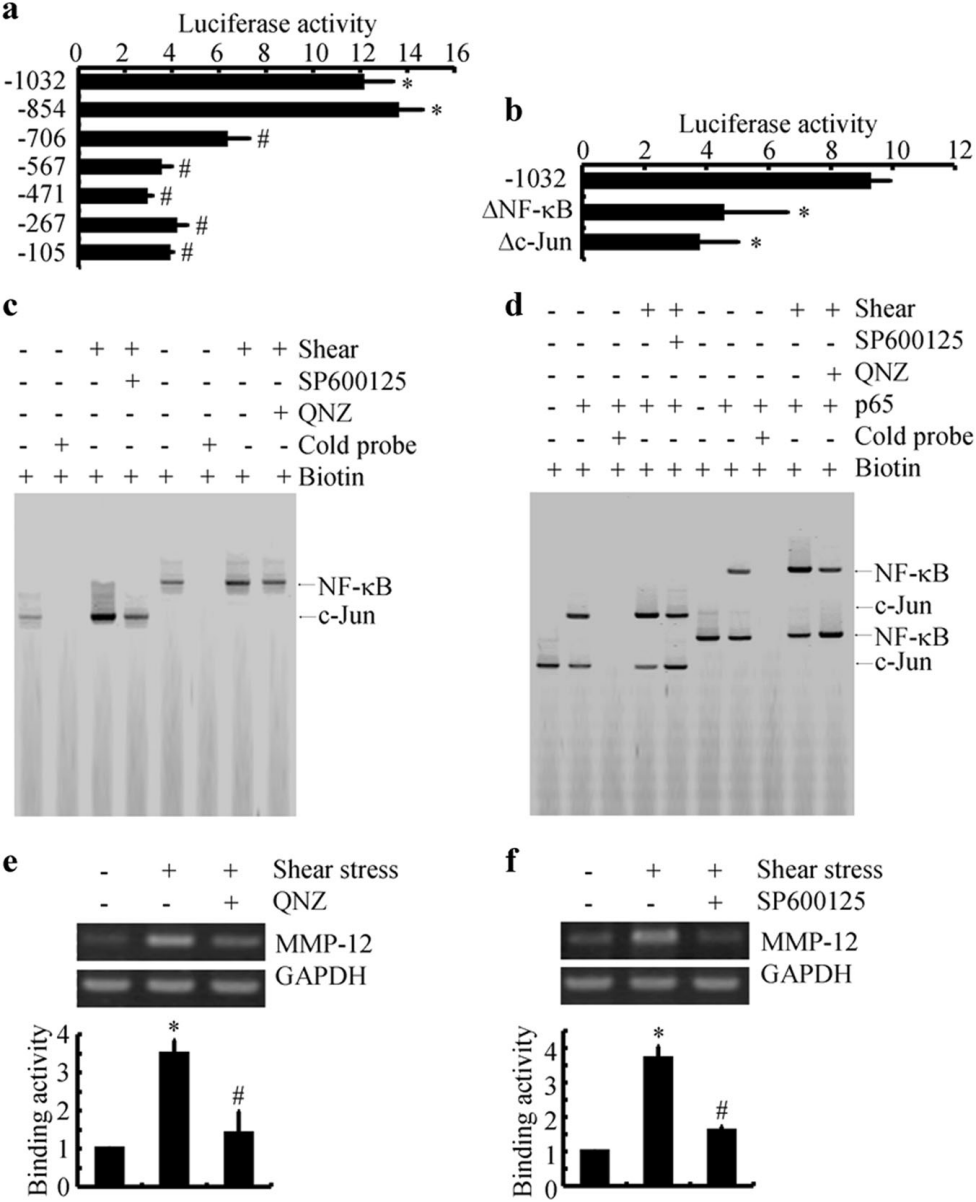
NF-κB and c-Jun are identified as the essential transcriptional factor for MMP-12 synthesis in shear-activated human chondrocytes. **a** and **b** T/C-28a2 cells were transfected with *mmp-12* promoter expression plasmids or mutation constructs before being subjected to fluid shear stress (20 dyn/cm^2^) for 6 h. Luciferase activities, normalized to *Renilla* luciferase activities, were measured using the Dual-Luciferase Reporter Assay Kit. **c** and **d** In select experiments, nuclear extracts were isolated, and NF-κB and c-Jun-specific DNA-protein complex formation was determined by EMSA. **e** and **f** In separate experiments, cross-linked chromatin was immunoprecipitated using an anti-p65 or anti-c-Jun antibody. In ChIP assays, the anti-RNA polymerase II antibody was used as a positive control. DNAs purified from both immunoprecipitated (IP) and preimmune (Input) specimens were subjected to qPCR amplification using primers for *mm-12* promoter genes. Data represent the mean ± SE of at least three independent experiments. * *p* *<* 0.05 compared with static or vehicle treatment control. ^#^ *p* < 0.05 with respect to shear stress or PGE_2_ treatment specimens

In view of our data suggesting that MMP-12 is modulated by the NF-κB and c-Jun pathways (Fig. 5a–d), we sought to confirm whether p65 and c-Jun transcriptionally mediated the effects of shear stress on stimulating the expression of MMP-12 in human chondrocytes. For this purpose, promoter analysis experiments were performed to identify the critical binding sites of NF-κB and c-Jun on the *mmp-12* promoter, which, in turn, regulate shear-induced MMP-12 synthesis. The results indicated that the binding sites of NF-κB and c-Jun resided between -567/-706 bp and -706/-854 bp upstream of the transcriptional start site, which are critical for the induction of *mmp-12* promoter activity in sheared chondrocytes (Fig. 6a). Mutations of NF-κB and AP-1 will result in a downregulation of the luciferase activity of *mmp-12* promoter constructs in shear-activated human chondrocytes (Fig. 6b). In addition, EMSA and ChIP assays were carried out to confirm the binding of phosphorylated NF-κB and c-Jun to their putative sites on the *mmp-12* promoter (Fig. 6c–f). Therefore, NF-κB and c-Jun are critical transcriptional factors for modulating shear-stimulated MMP-12 expression in T/C-28a2 chondrocytes.

### The key roles of COX-2 in inducing the expression of MMP-12 in articular cartilage of mice

**Fig. 7:**
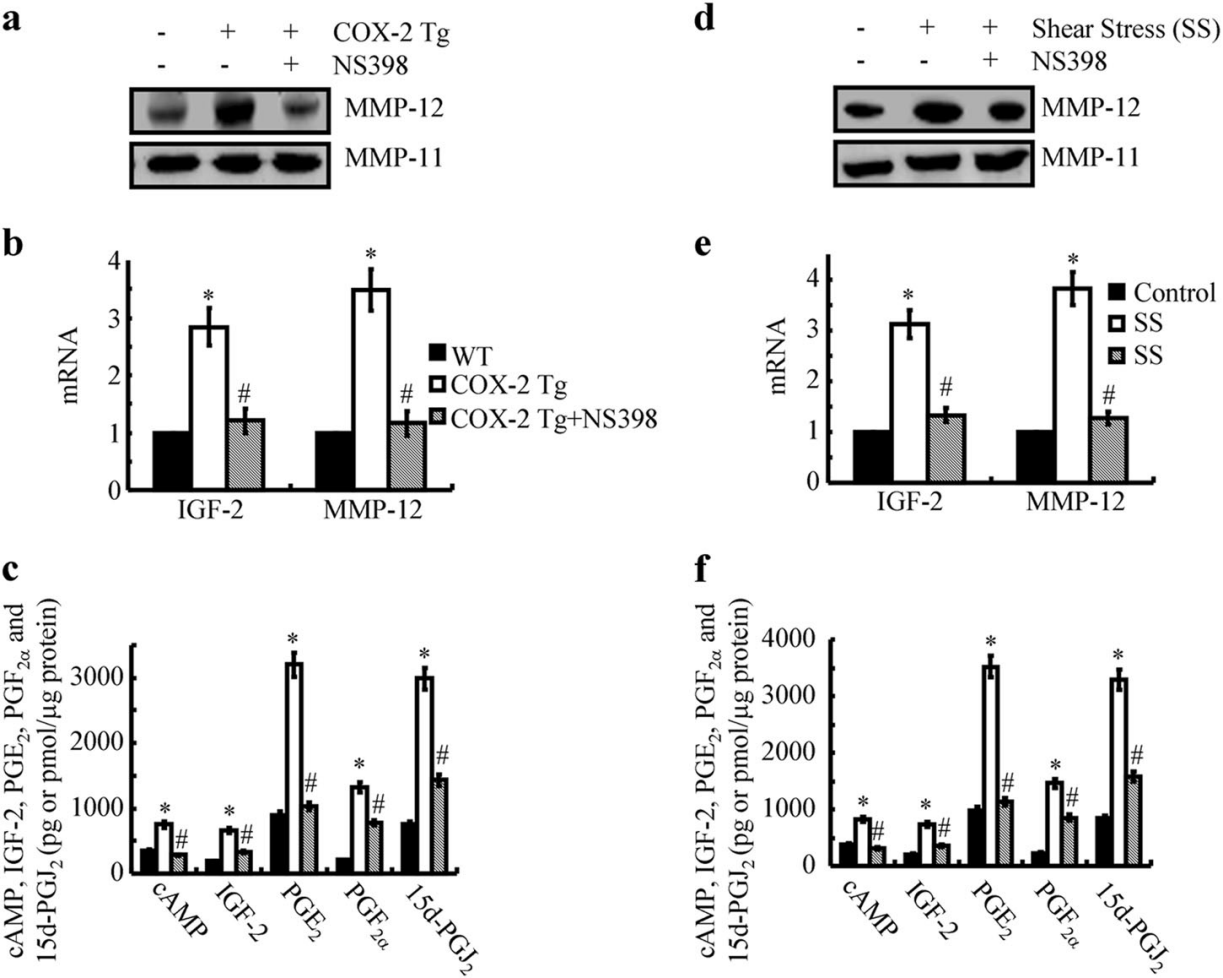
COX-2 plays pivotal roles in regulating MMP-12 expression in vivo. (**a**-**c**) COX-2 Tg mice at the age of 1-month-old were administered NS398 (2 mg/kg/d) for 2 months. **d**–**f** In select experiments, T/C-28a2 chondrocytes were subjected to fluid shear stress (20 dyn/cm^2^) or static conditions (0 dyn/cm^2^) in the absence or presence of NS398 (10 μM) for 6 h. The conditioned medium was collected and injected into the cavity of articular cartilage of WT mice. **a** and **d** MMP-12 mRNA and protein levels were determined by qRT-PCR and western blots, respectively. GAPDH and MMP-11 served as internal controls in qRT-PCR and western blots, respectively. **b**, **c**, **e**, **f** IGF-2 mRNA expression and secretion of cAMP, IGF-2, PGE_2_, and PGF_2α_ were determined using qRT-PCR and enzyme immunoassay kits, respectively. GAPDH and total protein in the medium served as internal controls in qRT-PCR and enzyme immunoassays, respectively. Data represent the mean ± SE of at least three independent experiments. *p *<* *0.05* with respect to static control. ^#^ *p* < 0.05 with respect to COX-2 Tg mice or shear stress treatment alone

To further evaluate the pivotal role of COX-2 in upregulating the expression of MMP-12 in vivo, COX-2 transgenic (Tg) mice were generated. In addition, RNA and protein were extracted from 3-month-old COX-2 Tg mice to determine the changes in gene and protein expression in articular tissue. The results demonstrated that COX-2 overexpression efficiently enhanced the production of cAMP, IGF-2, PGE_2_, PGF_2α_, and 15d-PGJ_2_ (Fig. 7b, c). More importantly, we found that the protein expression of MMP-12 was also elevated in the COX-2 Tg mice (Fig. 7a). Oral administration of NS398 (1 mg/kg/d) in COX-2 Tg mice for 2 months ameliorated the MMP-12 induction by decreasing the production of cAMP, IGF-2, PGE_2_, PGF_2_, and 15d-PGJ_2_ (Fig. 7a–c). Because high fluid shear stress (20 dyn/cm^2^) induces the secretion of cAMP, IGF-2, PGE_2_, PGF_2_, and 15d-PGJ_2_, we evaluated the ability of shear-conditioned medium on the expression of MMP-12 in wild-type (WT) mice. The results indicated that the injection of shear-conditioned medium to the articular cavity of WT mice clearly induced the mRNA expression of MMP-12 (Fig. 7d–f). In addition, NS398 treatment decreased the effects of shear-conditioned medium on stimulating MMP-12 expression in WT mice (Fig. 7d–f). These observations clearly indicated the pivotal roles of COX-2 in regulating the MMP-12 expression in mice.

### The involvement of cAMP, IGF-2, PGE_2_, PGF_2α_, and 15d-PGJ_2_ in regulating the expression of MMP-12 in articular cartilage in vivo

**Fig. 8:**
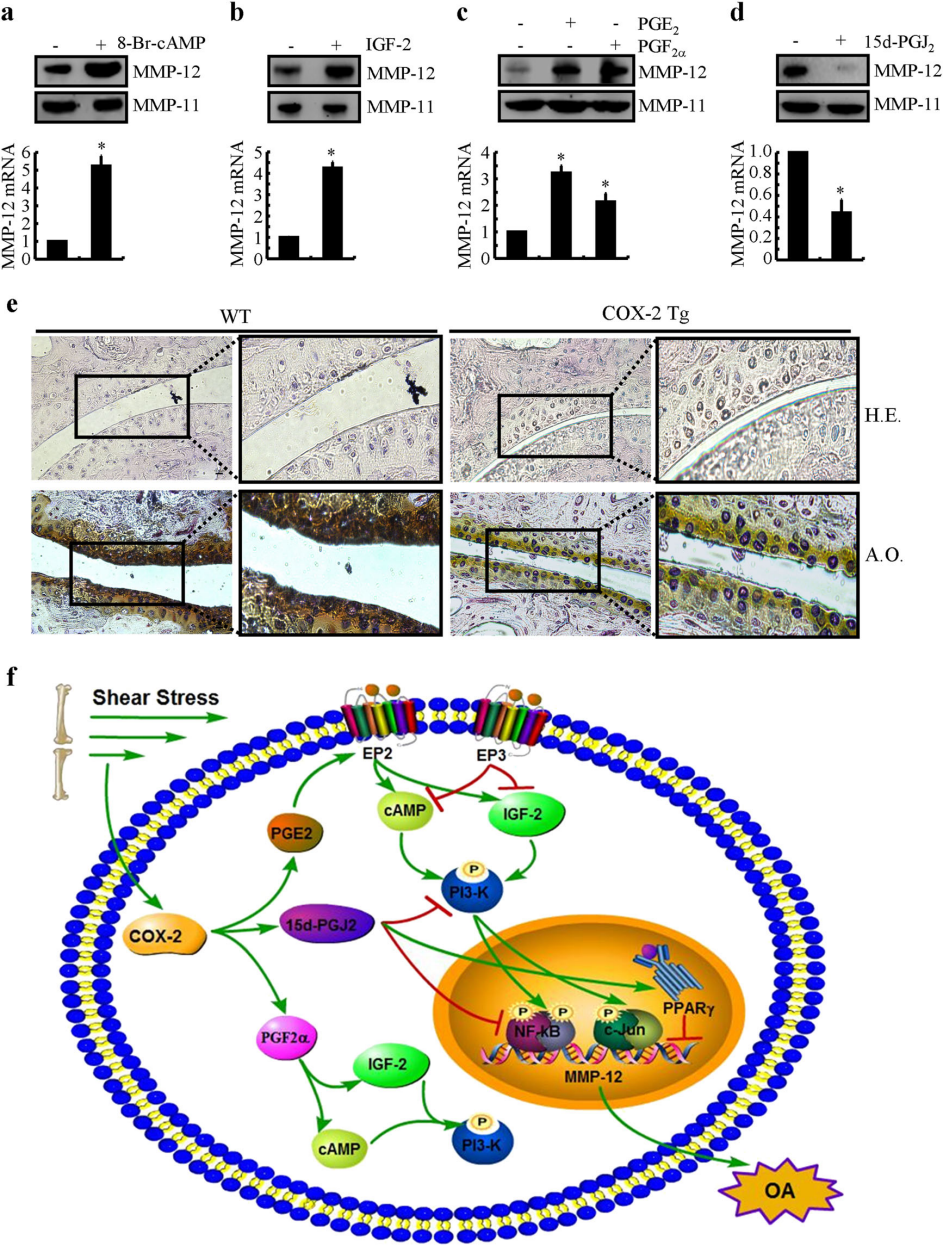
cAMP, IGF-2, PGE_2_, PGF_2α_, and 15d-PGJ_2_ upregulated the mRNA expression of MMP-12 in mice. **a** 8-Br-cAMP (2 μg/5 μl), (**b**) IGF-2 (10 ng/5 μl), (**c**) PGE_2_ (1 μg/5 μl) or PGF_2α_ (1 μg/5 μl) was injected into the cavity of articular cartilage of WT mice. **d** In select experiments, 15d-PGJ_2_ (1 μg/5 μl) was injected into the cavity of articular cartilage of COX-2 Tg mice. MMP-12 mRNA and protein levels were determined by qRT-PCR and western blots, respectively. GAPDH and MMP-11 served as internal controls in qRT-PCR and western blots, respectively. **e** Knee joint samples were dissected from 3-month-old mice, and alcian blue/hematoxylin and eosin and safranin O-fast green staining was performed. Data represent the mean ± SE of at least three independent experiments. *p *<* *0.05* with respect to PBS (-)-treated control. **f** Proposed cascade of signaling events regulates the progressive synthesis of MMP-12 in human chondrocytes with high fluid shear stress. High fluid shear stress induces the rapid and sustained expression of COX-2 and synthesis of PGE_2_ and PGF_2α_. The accumulation of PGE_2_ and PGF_2α_ proceeds via an EP2/EP3- and FPR-dependent cAMP and IGF-2 induction mechanism to increase the expression of MMP-12 at 6 h of shear stress exposure. In contrast, 15d-PGJ_2_ was produced after prolonged exposure to high fluid shear stress. The production of 15d-PGJ_2_ suppressed the expression of MMP-12 at 48 h of shear stress exposure. The roles of metabolic products of shear-induced COX-2 in regulating the mRNA and protein expressions of MMP-12 were finally verified in mice

Given the pivotal roles of COX-2 in the regulation of MMP-12 in vivo, we subsequently validated the effects of cAMP, IGF-2 PGE_2_, PGF_2α_, and 15d-PGJ_2_ on the expression of MMP-12 in vivo. For this purpose, we first injected 8-Br-cAMP (2 μg/5 μl), a stable analog of cAMP, into the articular cavity of WT mice. The results demonstrated that 8-Br-cAMP treatment significantly induced the expression of MMP-12 in the WT mice (Fig. 8a). Similarly, IGF-2 (10 ng/5 μl) injection clearly increased the expression of MMP-12 (Fig. 8b). As the downstream metabolic products of COX-2, we further injected PGE_2_ (1 μg/5 μl), PGF_2α_ (1 μg/5 μl), or 15d-PGJ_2_ (1 μg/5 μl) into the articular cavity of WT mice. As expected, the results demonstrated that the treatment of the WT mice with PGE_2_ and PGF_2_ induced the expression of MMP-12 (Fig. 8c). In contrast, 15d-PGJ_2_ injection (1 μg/5 μl) suppressed the expression of MMP-12 in the COX-2 Tg mice (Fig. 8d). To further validate the role of COX-2 signaling in OA development, experiments were carried out to stain the slices with alcian blue/hematoxylin and eosin and safranin O-fast green. Histological examination indicated that hypertrophic chondrocytes localized at the surface of the articular cartilage, which indicates early osteophyte formation in 3-month-old mice. Morphological analysis suggested a significant reduction in the articular cartilage area in 3-month-old COX-2 mice (Fig. 8e). These findings showed a potential OA-like phenotype in 3-month-old COX-2 Tg mice compared to WT controls. Taken together, our findings suggest that cAMP, IGF-2 PGE_2_, PGF_2α_, and 15d-PGJ_2_ mediated the effects of shear stress on stimulating MMP-12 expression in vivo during the course of OA development.

### Deciphering the mechanisms of MMP-12 upregulation by shear stress in the COX-2 and PGs-dependent pathways potentially provide the therapeutic targets for treating OA

Using in vitro techniques of high fluid shear stress, we show that high fluid shear stress elevated the expression of COX-2^31^ and the synthesis of PGE_2_, PGF_2α_, and 15d-PGJ_2_. The synthesis of PGE_2_ and PGF_2α_ is responsible for MMP-12 activation via EP2/EP3-dependent and FPR-dependent mechanisms of activating cAMP and IGF-2. During this process, PI3-K plays critical roles in mediating the stimulatory effects of cAMP and IGF-2 on MMP-12 expression via an NF-κB- and c-Jun-transactivating mechanism. More interestingly, 15d-PGJ_2_ suppressed the expression of MMP-12 via the PPARγ pathway, the activity of which decreased during the late stage of OA (Fig. 8f).

By identifying the signaling pathways of MMP-12 induction by high fluid shear stress, our data potentially provide the therapeutic targets for treating OA. More specifically, at least four potential therapeutic targets might be identified through deciphering the current signaling pathways, including (1) COX-2; (2) PGs receptors, such as EP2, EP3, and PPARγ; (3) signaling molecules, such as PI3-K, NF-κB, and c-Jun; and (4) MMP-12. By identifying these targets, therapeutic drugs might be potentially established for future clinical practice, such as (1) inhibitors of COX-2, such as celecoxib, rofecoxib, and other NSAIDs; (2) agonists or antagonists of the PGs receptors, such as AH6809, sulprostone, and GW9662; (3) pharmacological agents, such as LY294002, wortmannin, QNZ, and SP600125; and (4) inhibitors of MMP-12, such as MMP408. On the basis of these pharmacological interventions, OA might be targeted therapy.

## Discussion

High fluid shear stress has recently been identified as being involved in disrupting the articular cartilage, thereby adversely affecting chondrocyte function and precipitating OA.^7^ A series of investigations support the notion that high fluid shear stress on chondrocytes recapitulates the hallmark of OA by inducing the expression of proinflammatory cytokines and PGs, which, in turn, exacerbate the disease by activating MMPs.^8,9^ In view of the involvement of MMP-12 upregulation in the pathogenesis of OA, we herein identify the mechanism of MMP-12 elevation in shear-stimulated human T/C-28a2 chondrocytes.

MMP-12 was found to be upregulated in the subchondral bone of OA rats.^7^ In addition, MMP-12 expression was positively correlated with the age and body mass index of OA patients.^23^ MMP-12 expression was also upregulated in the Zn^2+^-ZIP8-MTF1-regulated pathogenesis of OA.^24^ In line with these observations, we further showed that MMP-12 expression was stimulated in shear-activated human chondrocytes. Notably, the marked induction of MMP-12 during the course of OA progression was caused, at least in part, by excessive mechanical loading, particularly high fluid shear stress.

To decipher the underlying mechanism, we investigated the roles of COX-2 and its metabolic products, such as PGE_2_ and PGF_2α_, in the regulation of MMP-12. It has been reported that (a) high fluid shear stress activates COX-2 and the production of PGs at various time points;^3–6^ (b) PGE_2_ and 15d-PGJ_2_ are involved in regulating the expression and enzymatic activity of MMPs;^8,9^ and (c) fibroblast growth factor 2 (FGF-2) is critical for mediating the effects of PGE_2_ and 15d-PGJ_2_ on regulating the MMP expression in shear-stimulated chondrocytes.^8,9^ Based on these previous studies, we further characterized the roles of COX-2 and PGs in MMP-12 synthesis. Our results demonstrated that PGE_2_ and PGF_2α_ are responsible for upregulating the expression of MMP-12 in shear-activated human chondrocytes.

As PGE_2_ and PGF_2α_ are capable of inducing the expression of MMP-12, we further found that PGE_2_ and PGF_2α_ have the ability to stimulate the activity of PI3-K-dependent NF-κB and c-Jun in the EP2/EP3- and FPR-dependent cAMP pathways. Our previous studies have shown that EP2 and EP3 mediated the stimulatory effects of PGE_2_ on the activity of PI3-K-dependent NF-κB and c-Jun by inducing the formation of cAMP, although we did not extend this pathway to MMP-12 regulation in shear-activated human chondrocytes.^3,5,8,9^ In addition, Collins et al.^32^ also reported that PGF_2α_ treatment markedly enhanced the levels of cAMP in isolated human colonic crypts. In contrast, Khan et al.^33^ reported that PGF_2α_ inhibits cAMP accumulation in rat corpora lutea. These findings indicated that the differences in PGF_2α_-mediated regulation of cAMP production may be ascribed to different cell types. Because there is no additional evidence showing the relationship between PGF_2α_ and cAMP, we treated human chondrocytes with PGF_2α_. These results demonstrated that PGF_2α_ markedly increased the production of cAMP in a PGF_2α_ receptor (FPR)-dependent manner.

Apart from cAMP, we further found that IGF-2 is located downstream of PGE_2_ and PGF_2α_ and is responsible for the synthesis of MMP-12 in shear-stimulated human chondrocytes. Although there is no direct evidence showing the regulatory pathway between PGE_2_ and IGF-2, Hilal et al.^34^ reported that both PGE_2_ formation and IGF-2 signaling regulate the expression of parathyroid hormone receptor in human OA osteoblasts. Unfortunately, they did not extend their studies to further identify the relationship between PGE_2_ and IGF-2. More closely, Di Battista et al.^35^ showed that PGE_2_ stimulates IGF binding protein-4 (IGFBP4) expression in cultured human articular chondrocytes. In addition, they emphasized that PGE_2_ induces the synthesis of collagen and proteoglycan in articular cartilage via an autocrine feedback loop involving IGF-1,^35^ which is responsible for the regeneration of articular cartilage.^36^ In addition, Hakeda et al.^37^ reported that PGF_2α_ can stimulate the proliferation of clonal OA MC3T3-E1 cells via the upregulation of IGF-1. Bukar et al.^38^ further showed that PGF_2α_ treatment stimulated the plasma expression of IGF-1 in goats. Consistent with these observations, Grimes et al.^39^ showed that PGF_2α_ can upregulate the expression of IGFBP3 in luteinized granulose cells. Similarly, our data extended previous studies to show that PGE_2_ and PGF_2α_ exerted their biological function via the downstream targets of IGF-2 in shear-activated human chondrocytes.

cAMP accumulation augments MMP-12 expression in human chondrocytes via the PI3-K and AKT signaling pathways. Consistent with these observations, our previous work has demonstrated that cAMP formation can stimulate the PI3-K and AKT pathway.^3,5,6,8,9^ These observations were further confirmed by the fact that cAMP synthesis is critical for the synthesis of target genes in response to PGE_2_ treatment in human chondrocytes.^8,9^ Consistent with these observations, we further found that IGF-2 is another molecule important for stimulating the PI3-K-, NF-κB-, and c-Jun-dependent signaling pathways, which are responsible for MMP-12 expression in shear-activated chondrocytes. In concert with our findings, IGF-2 is also reported to activate the pathways of PI3-K during chondrogenesis.^40^ Furthermore, IGF-2 is critical for activating MMP-12 in human chondrosarcomas.^26^ IGF-1, which belongs to the same protein family, has also been reported to be an upstream component of the PI3-K and AKT pathways in various experimental models.^41^ However, Arvisais et al.^42^ reported that PGF_2α_ represses the IGF-1-stimulated activation of the PI3-K and AKT signaling pathways by stimulating the expression of ERK in the corpus luteum. This difference may be attributed to the divergent responses of distinct cell types. Moreover, we could not confirm a pivotal role for ERK in human chondrocytes.

Given previous studies,^8,9^ we continue to discuss the roles of downstream transcriptional factors, including NF-κB and c-Jun, in the synthesis of MMP-12. NF-κB and c-Jun have been indicated in regulating the expression of MMP-12 in human A431 and U937 cells.^43,44^ By the pharmacological interventions of inhibitors, we reported the involvement of NF-κB and c-Jun in inducing the expression of MMP-12 and OA pathogenesis. Because the inhibition of NF-κB and c-Jun activity blocks forskolin- and IGF-2-induced MMP-12 synthesis, we propose that the binding of phosphorylated NF-κB and c-Jun to their putative sites on the *mmp-12* promoter is critical to the induction of MMP-12 expression in human chondrocytes. However, we cannot exclude the possibility that binding of phosphorylated c-Jun to its putative AP1 binding sites on the *mmp-12* promoter might also contribute to MMP-12 expression.

Previous studies have demonstrated that mechanical stimuli may transduce their intracellular signaling pathways via the activation of L-PGDS and the release of endogenous PGD_2_ and its metabolite, 15d-PGJ_2_, in human chondrocytes.^45^ As a consequence, 15d-PGJ_2_ was reported to attenuate or block the induction of MMPs in cytokine-stimulated chondrocytes.^46^ In line with these studies, we further found that 15d-PGJ_2_ inhibited the shear-induced expression of MMPs in shear-stimulated human chondrocytes.^8,9^ Based on these observations, we investigated the mutual interaction between 15d-PGJ_2_ and MMP-12 synthesis. Our results demonstrate that both exogenous and endogenous 15d-PGJ_2_ can suppress MMP-12 expression by activating PPARγ. In concert with our data, Lian et al.^47^ indicated that the treatment of H441 cells with PPARγ ligands, including 9-HODE and ciglitazone, inhibits MMP-12 promoters. Furthermore, our previous studies demonstrated that 15d-PGJ_2_ suppressed the mRNA expression of MMP-12 in sheared chondrosarcomas.^26^ The actions of 15d-PGJ_2_ on the expression of MMP-12 caused the decrease in MMP-12 after prolonged shear stress exposure.

More interestingly, PGE_2_ and 15d-PGJ_2_ showed antagonistic effects on not only the activity of MMP-12 but also the inflammation of OA. For example, IL-6 has been reported to be upregulated by PGE_2_ treatment in T lymphocytes.^48^ Similarly, the expressions of IL-1β, IL-6, and IL-10 are markedly induced in OA cartilage and shear stress-activated cells, which indicates that OA or fluid shear stress likely stimulates the expression of IL-1β, IL-6, and IL-10 via the COX-2/PGE_2_ signaling pathways.^7^ The opposite effects of 15d-PGJ_2_ on the PGE_2_-induced activity of MMP-9^8^ might imply antiinflammatory effects on OA occurrence and progression. In addition, the secretion of many proinflammatory cytokines, such as IL-1β, IL-6, IL-12(p70), IL-12(p40), TNF-α, and NO, are inhibited by 15d-PGJ_2_.^7^

Given the pivotal roles of COX-2 and its metabolic products, such as PGE_2_ and PGF_2α_, in the pathogenesis of OA, it is easy to speculate that nonsteroidal antiinflammatory drugs (NSAIDs) are the main therapeutic drugs for treating OA patients. As specific inhibitors of COX-2, celecoxib and rofecoxib were developed to treat OA, which improved the pathogenesis of OA in cartilage.^49^ Furthermore, licofelone, an inhibitor of COXs, has been used to treat OA by suppressing the activity of MMP-13.^50^ In agreement with this report, we further found that NS398 treatment clearly inhibited the pathogenesis of OA by suppressing MMP-12 expression. Thus, it is clear that inhibitors of COX-2 exert their effects on suppressing the occurrence and progression of OA by attenuating the activity of MMPs. More importantly, the treatment of OA with COX-2 inhibitors together with antagonists of proinflammatory cytokines and MMPs will depend on further mechanistic investigations.

In conclusion, MMP-12 is clearly induced in human OA tissues compared to normal bone controls. To decipher the mechanisms of MMP-12 upregulation, high fluid shear stress was applied to human chondrocytes. We found that high fluid shear stress induced the rapid and sustained synthesis of MMP-12 via cAMP and IGF-2 and an EP2/EP3-dependent and FPR-dependent mechanism during the early stage of OA. More interestingly, 15d-PGJ_2_ suppressed the stimulatory effects of fluid shear stress on the MMP-12 expression during the late stage of OA. These findings are important for understanding the induction of MMP-12 during the course of OA development and for improving therapeutic strategies for combating OA.

## Electronic supplementary material

Supplemental Information
