# Supplementary material for: The roles of prostaglandin F2 in regulating the expression of matrix metalloproteinase-12 via an insulin growth factor-2-dependent mechanism in sheared chondrocytes
Source: Signal Transduct Target Ther. 2018 Nov 23;3:27. doi: 10.1038/s41392-018-0029-2 (PMC6261940; doi:10.1038/s41392-018-0029-2)
Supplement: Supplementary file 1 — Supplemental Information [file 41392_2018_29_MOESM1_ESM.doc]

**Supplemental Information**

**The roles of prostaglandin F_2_ in regulating the expression of matrix metalloproteinase-12 via an insulin growth factor-2-dependent mechanism in sheared chondrocytes**

Pei-Pei Guan^1^, Wei-Yan Ding^1^, Pu Wang^1, *^

^1^College of Life and Health Sciences, Northeastern University, Shenyang 110819, P. R. China

**Running title:** Shear-induced expression of MMP-12

* To whom correspondence should be addressed:

Pu Wang, Ph.D.

College of Life and Health Sciences

Northeastern University

No. 3-11. Wenhua Road

Shenyang, P. R. China 110819

Tel: +86-24-83656109

Email: wangpu@mail.neu.edu.cn

**Materials and Methods**

***Luciferase promoter constructs***

A 1062-base pair (bp) *mmp-12* promoter construct, which contains the 5′-flanking region of the human MMP-12 gene from -1032 to +30 relative to the transcription start site, was generated from human genomic DNA. The construct was created using specifically designed forward (5′- CCGAGCTCTT**ACGCGT**TTAACACACTCTTACGTGCAC-3′) and reverse (5′- GATCGCAGAT**CTCGAG**CTGCAGGAGCAGTATTAGAAG-3′) primers, which included Mlu1 and Xho1 restriction sites (underlined) at their 5′ ends and 3′ ends, respectively. The amplicon was inserted upstream of the luciferase reporter gene in the pGL3-basic vector (Promega). Different lengths of truncations were generated using primers that incorporated the relevant restriction enzyme sites (underlined) to enable proper orientation upstream of the luciferase reporter. The following truncated constructs were generated using the indicated primers: the -854/+30 bp construct, forward 5′-CCGAGCTCTT**ACGCGT**GTGCTTAAATATTCTCTATTGAGTAAGAG-3′; the −706/+30 bp construct, forward 5′- CCGAGCTCTT**ACGCGT**CTACCAACGCTAGACAATCTAG-3′; the −567/+30 bp construct, forward 5′-CCGAGCTCTT**ACGCGT**GGAATAAACAATGAAGTCGATGTTAC-3′; the -471/+30 bp construct, forward 5′-CCGAGCTCTT**ACGCGT**CACTCATTTACTCCAGGAAAAGG-3′; the -267/+30 bp construct, forward 5′-CCGAGCTCTT**ACGCGT**GTAGAGGCTTATTTATCATTTTGAAGG-3′; and the -105/+30 bp construct, forward 5′-CCGAGCTCTT**ACGCGT**CATATTCACAGAACCCGGAC-3′. All constructs were sequenced and confirmed to be identical to the sequence of the *mmp-12* promoter. For the NF-κB construct, site-directed mutagenesis was used to alter the sequence between −670 and −680 bp from 5′-ta**ggg**aaagtt-3′ to 5′-ta**ttt**aaagtt-3′ to block the binding between NF-κB and the *mmp-12* promoter. Similarly, the c-Jun construct was also altered between -795 and -801 bp from 5′-Tag**g**tca-3′ to 5′-Tag**t**tca-3′. All constructs were confirmed by DNA sequencing. The pRL-SV40 vector that encoded the *Renilla* luciferase gene was obtained from Promega Corporation (Madison, WI, USA).

***Promoter activity assays***

The activities of Firefly and *Renilla* luciferase were determined using the Dual-Luciferase Report Assay kit (Promega, Madison, WI, USA). In brief, the cells were lysed with 20 μl 1×passive lysis buffer at room temperature for 15 min on a rocking platform after 44-48 h of transfection. The lysates were subsequently assayed for luciferase activity by the Dual Luciferase Reporter Assay System (Promega, Madison, WI, USA). Briefly, 20 μl of lysate was mixed with 80 μl of luciferase assay reagent (LARII) in 96 well plates. The plates were then rapidly placed in the counter. The activity of Firefly luciferase was measured at 640 nm. Then, 80 μl of stop/Glo substrate was added to the well to measure the *Renilla* luciferase at 480±20 nm. *Renilla* luciferase controls were used as the baseline for calculating the activities of Firefly luciferase. Data were calculated and expressed as ratios of shear to static normalized firefly luciferase activity.

***EMSA***

EMSAs were performed according to a commercialized EMSA kit (LightShift Chemiluminescence EMSA kit; Pierce Protein Biology, Shanghai, China). Moreover, 5’-biotinylated oligonucleotide probes that contained the NF-κB (5’-T**tagggaaagtt**T-3’) and c-Jun (5’-CC**Taggtca**A-3’) cis-element present on the *mmp-12* promoter were synthesized. In brief, nuclear extracts (1-2 μg) were incubated with 20 fmol of biotinylated and double-stranded probes for NF-κB or c-Jun in 10× binding buffer (supplemented with 50 ng of poly (dI-dC), 2.5% glycerol (ml: ml), 0.05% Nonidet P-40 (ml: ml), 5 mM MgCl_2_, and 0.25 mg of bovine serum albumin) for 30 min on ice. This solution mixture was finally adjusted to 20 μl with distilled water. For competing binding, nuclear extracts were incubated with a 200-fold excess of unlabeled (cold) probe prior to the addition of the biotinylated probe. For the supershift assays, the nuclear extracts were initially incubated with an anti-p65 or c-Jun antibody for 30 min on ice. The mixtures were then incubated with a specific probe for NF-κB or c-Jun for an additional 30 min on ice. To exclude the possibility of nonspecific binding, a random 5’-biotinylated probe (5’-TGGTATGTATGAA-3’; 5’-CGCTAATGCA-3’) designed using a random sequence generator was also prepared for the shift and supershift assays. The reaction products of the protein-DNA complexes were electrophoresed in a native 6% polyacrylamide retardation gel in 0.5× Tris borate-EDTA running buffer at 10 mA for 1 h. The gels were then transferred to a nylon membrane (Pierce Protein Biology, Shanghai, China), which was visualized by the LightShift Chemiluminescence kit (Pierce Protein Biology, Shanghai, China).

***ChIP assay***

As previously described, the ChIP assay was exerted according to the manufacturer’s instructions of the EZ ChIP kit (Upstate Biotechnology, Inc. Waltham, MT, USA).^1,2^ In brief, 1×10^6^ cells were incubated and cross-linked in 1% formaldehyde for 10 min. The cells were then resuspended in 200 μl of CHIP/SDS lysis buffer after washing with PBS (-). The cell lysates were further incubated on ice for 10 min. To fragment DNA, 200 to 1000 bps DNAs were obtained by sonication to shear the chromatin. The supernatant was transferred to a fresh tube and mixed with 1.8 ml of ChIP dilution buffer after centrifugation. 40 μl of the supernatant was then transferred to another Eppendorf tube as 2% input. To avoid nonspecific binding, the diluted supernatants were precleared with 50 μl of protein G (Upstate Biotechnology) for 30 min at 4 ºC with rotation. The protein G was then removed after centrifugation. The supernatants were further added with 5 μl of anti-NF-κB or c-Jun for 18 h at 4 ºC with rotation. To bind immune complex, 50 μl of protein G was added to the sample and incubated for 1 h at 4 ºC with rotation. The protein-DNA complex was incubated with DNA extraction buffer to purify the DNA that bound to the immune complex using routine steps. The purified DNA was resuspended in 50 μl H_2_O and used in the subsequent PCR analysis. The PCR primers for the MMP-12 promoter are as follows: NF-κB, F- ggcagatagattccagaggc, R- gttgtaccggctcagatcct and c-Jun, F- ctacctttggaattttattccctg, R- gattgtctagcgttggtagg. The PCR parameters were 95 ºC for 5 min; 30 cycles of 95 ºC 15 s, 58 ºC 30 s, and 68 ºC 1 min; followed by extension at 77 ºC 5 min.^2^ The ratio of the binding activity was calculated by the following equation:

Ratio=$\frac{\Delta O.D.(MMP-12_{Control}-MMP-12_{Treatment})}{\Delta O.D.(GAPDH_{Control}-GAPDH_{Treatment})}$

***Transgenic mice***

Wild-type (WT) and COX-2 transgenic (Tg) mice were obtained from The Jackson laboratory (Bar Harbor, ME, USA). Mice at the age of 1 month were treated with a COX-2 inhibitor, NS398 (1 mg/kg/d), for 2 months prior to euthanasia. In separate experiments, the 8-Br-cAMP (2 μg/5 μl), IGF-2 (10 ng/5 μl), PGE_2_ (1 μg/5 μl), PGF_2α_ (1 μg/5 μl) or 15d-PGJ_2_ (1 μg/5 μl) was injected into the articular cavity of the mice prior to tissue collection.^3^ In brief, the pharmacological agents were intra-articularly injected with an insulin syringe (0.5 ml monoject (29G), Changzhou Jinlong Medical Plastic Appliance Co., LTD. China) through the patellar ligament into the right knee joint. Notably, stretching of the rear-leg facilitated the intra-articular injection. The cartilages of the mice in the different groups were collected after euthanasia as previously described.^1^ Following the intra-articular injection, the mice were sacrificed at the indicated times by inhaling excess ether. The skin was then removed from the legs, and the knee joints were dissected from the associated muscle. Knee joints were briefly dipped in a 5% aqueous solution of polyvinyl alcohol, followed by fixation in neutral buffered formalin saline. The immobilized joints were decalcified with formic acid at 4°C prior to embedding in paraffin. Mid-sagittal serial slices (5 μm thick) were sectioned and stained with MMP-12 specific antibody, hematoxylin and eosin (H&E) or alcian blue and safranin O-fast green (A&O). In select experiments, the knee joints were snap frozen at −180 °C in liquid nitrogen. The knee joints were grinded by mortar in liquid nitrogen. The powders of the knee joints were then submerged in trizol or RIPA buffer to extract total mRNA and protein. The mRNA and protein were subsequently stored at -80°C until use for qRT-PCR, ELISA or western blots.

***Immunohistochemistry (IHC)***

Joint cartilage tissues were collected from 3-month-old wild type or COX-2 Tg mice. Paraffin sections (5 μm thick) were cut by radial microtomes after decalcification (Leica, CM2235, Germany). MMP-12 was immunostained with an immunohistochemical staining kit according to the manufacturer’s instructions (Invitrogen, Carlsbad, CA, USA). In selected experiments, serial sections were stained with Alcian blue and Safranin O-fast green or hematoxylin and eosin for morphologic analysis.

***Animal committee***

All animals were handled according to the care and use of medical laboratory animals (Ministry of Health, Peoples Republic of China, 1998), and all experimental protocols were approved by the Laboratory Ethics Committees of China Medical University [SYXK(Liaoning, China)2013-0007, Deliang Wen].

**References**

1 Guan, P. P. *et al.* The role of cyclooxygenase-2, interleukin-1beta and fibroblast growth factor-2 in the activation of matrix metalloproteinase-1 in sheared-chondrocytes and articular cartilage. *Sci. Rep.* **5**, 10412 (2015).

2 Guan, P. P. *et al.* By activating matrix metalloproteinase-7, shear stress promotes chondrosarcoma cell motility, invasion and lung colonization. *Oncotarget* **6**, 9140-9159 (2015).

3 Kehoe, O., Cartwright, A., Askari, A., El Haj, A. J. & Middleton, J. Intra-articular injection of mesenchymal stem cells leads to reduced inflammation and cartilage damage in murine antigen-induced arthritis. *J. Transl. Med.* **12**, 157 (2014).
